# Targeted Energy Intake Is the Important Determinant of Clinical Outcomes in Medical Critically Ill Patients with High Nutrition Risk

**DOI:** 10.3390/nu10111731

**Published:** 2018-11-11

**Authors:** Chen-Yu Wang, Pin-Kuei Fu, Chun-Te Huang, Chao-Hsiu Chen, Bor-Jen Lee, Yi-Chia Huang

**Affiliations:** 1 Department of Critical Care Medicine, Taichung Veterans General Hospital, Taichung 40705, Taiwan; chestmen@gmail.com (C.-Y.W.); yetquen@gmail.com (P.-K.F.); huangchunte@gmail.com (C.-T.H.); borjenlee@gmail.com (B.-J.L.); 2 Department of Nursing, Hung Kuang University, Taichung 43302, Taiwan; 3 College of Human Science and Social Innovation, Hung Kuang University, Taichung 43302, Taiwan; 4 School of Chinese Medicine, China Medical University, Taichung 40402, Taiwan; 5 Department of Food and Nutrition, Taichung Veterans General Hospital, Taichung 40705, Taiwan; hsiu@vghtc.gov.tw; 6 School of Medicine, Chung Shan Medical University; Taichung 40201, Taiwan; 7 Department of Nutrition, Chung Shan Medical University, Taichung 40201, Taiwan; 8 Department of Nutrition, Chung Shan Medical University Hospital, Taichung 40201, Taiwan

**Keywords:** nutrition risk, energy intake, mortality, critically ill patients, medical ICU

## Abstract

The clinical conditions of critically ill patients are highly heterogeneous; therefore, nutrient requirements should be personalized based on the patient’s nutritional status. However, nutritional status is not always considered when evaluating a patient’s nutritional therapy in the medical intensive care unit (ICU). We conducted a retrospective cross-sectional study to assess the effect of ICU patients’ nutrition risk status on the association between energy intake and clinical outcomes (i.e., hospital, 14-day and 28-day mortality). The nutrition risk of critically ill patients was classified as either high- or low-nutrition risk using the modified Nutrition Risk in the Critically Ill score. There were 559 (75.3%) patients in the high nutrition risk group, while 183 patients were in the low nutrition risk group. Higher mean energy intake was associated with lower hospital, 14-day and 28-day mortality rates in patients with high nutrition risk; while there were no significant associations between mean energy intake and clinical outcomes in patients with low nutrition risk. Further examination of the association between amount of energy intake and clinical outcomes showed that patients with high nutrition risk who consumed at least 800 kcal/day had significantly lower hospital, 14-day and 28-day mortality rates. Although patients with low nutrition risk did not benefit from high energy intake, patients with high nutrition risk are suggested to consume at least 800 kcal/day in order to reduce their mortality rate in the medical ICU.

## 1. Introduction

The clinical conditions of critically ill patients are highly heterogeneous. It has been recently shown that not only malnutrition was associated with poor outcome [1] but also that energy expenditure was related with adenosine triphosphate concentration [2]. Therefore, nutritional intake should be tailored to the individual needs of the patient. Previous studies showed no outcome differences among trophic feeding, permissive under feeding and full caloric feeding among critically ill patients [3,4]. A study with a large sample size conducted by Arabi et al. [5] also found no significant difference in 90-day mortality of critically ill patients between permissive underfeeding (*n* = 448) and standard enteral feeding (*n* = 446) at 7 centers. However, the lack of nutrition risk stratification might explain why there were no differences in clinical outcome among studies using different feeding strategies. According to the ASPEN/SCCM guidelines, nutritional therapy for critically ill patients should take nutritional status into account which can be assessed using the Nutritional Risk Screening 2002 or the Nutrition Risk in the Critically Ill (NUTRIC) score [6,7,8].

The NUTRIC score is the first nutrition risk assessment tool for critically ill patients and consists of 6 factors: age, acute physiology and chronic health evaluation II (APACHE II) score, baseline simplified organ failure assessment score, number of comorbidities, days in hospital to ICU admission and interleukin-6 (IL-6) level [8]. Since IL-6 is rarely available in the intensive care unit (ICU), a modified NUTRIC score (mNUTRIC score) without IL-6 level was used as a more practical alternative [9]. Patients were defined as being at “high nutrition risk” with a NUTRIC score or a mNUTRIC score ≥5 [10]. The mNUTRIC score has been shown to be well correlated with clinical outcomes (i.e., 28-day mortality) [8,11,12]. Patients with high nutrition risk assessed by mNUTRIC score had reduced mortality if they received higher energy intakes in the ICU, although this trend was not observed in patients with low nutrition risk [9,11,13]. Moreover, in a post-hoc analysis of the PermiT trial, there was no association between feeding strategy (permissive underfeeding vs. standard feeding group) and mortality, regardless of low or high nutrition risk [14].

The results of studies assessing the relationship between energy intake and clinical outcomes in patients with different nutrition risk remain inconsistent. In order to clarify this clinical issues, we conducted a study to assess the association between energy intake and clinical outcomes (i.e., hospital mortality, 14-day and 28-day mortality) in medical ICU patients with high and low nutrition risk. We hypothesized that associations between energy intake and clinical outcomes might be observed in patients with different nutrition risk.

## 2. Materials and Methods

### 2.1. Study Design and Sample Size Calculation

This study employed a retrospective, cross-sectional design. The study was conducted in the medical ICUs of Taichung Veterans General Hospital (TCVGH) in Taichung, a tertiary medical center located in central Taiwan. This study was approved by the Institutional Review Board of TCVGH (IRB No. CE17191B). The data in this study were retrospectively obtained from medical charts and anonymized, so the requirement for informed consent was waived by the Institutional Review Board of TCVGH.

The size of the patient sample was based on the requirements for the detection of a significant correlation coefficient of 0.2 between mortality and energy intakes with a power of 90% and a 2-sided test with an α of 0.05. The required sample size was therefore a minimum of 259 subjects.

### 2.2. Patients

We retrospectively enrolled patients by reviewing medical charts from September 2015 to August 2016. We included patients who were older than 20 years old, had mechanical ventilator support and stayed in the ICU for more than 48 hours. The exclusion criteria included upper gastrointestinal bleeding, or any periods of nil per os in the first 7 days after ICU admission.

### 2.3. Data Collection and Outcomes

Clinical data collection included patients’ age, gender, height, weight, use of propofol, daily energy intake from glucose infusion and parenteral or enteral support from the day of ICU admission to the 7th day or the day of discharge from the ICU, diagnosis, APACHE II score, sequential organ failure assessment score, days in hospital to ICU admission, number of comorbidities, length of ventilator dependence, length of hospital and ICU stays and survival days. Body mass index (BMI) was calculated from height and weight. The mNUTRIC score was then calculated using the available data and patients were accordingly assigned the high nutrition risk (5–9 score) and low nutrition risk (0–4 score) groups. The amount of energy intake is presented in the results as the average of energy intake within the first 7 days’ stay in the ICU.

We compared the primary outcome of hospital mortality between the high and low nutrition risk groups. Secondary outcomes of 14-day and 28-day mortality were also compared between groups.

### 2.4. Statistical Analysis

All data were analyzed using the SAS statistical software package (version 9.4; Statistical Analysis System Institute Inc., Cary, NC, USA). The Shapiro-Wilk test was tested for the normality of sample distribution. In order to compare the groups for significance, continuous variables were compared using the Student’s *t*-test or Mann-Whitney Rank Sum test. Categorical variables were compared using the Chi-square test or Fisher’s exact test. Multivariate logistic regression was used to estimate the odds ratios and 95% confidence intervals for hospital, 14-day and 28-day mortality. Statistical results were considered statistically significant at *p* < 0.05.Values presented in the text are means ± standard deviation (SD), the frequency, or percentage rates.

## 3. Results

Table 1 shows the demographic characteristics, clinical outcomes and energy intakes in critically ill patients. A total of 742 patients (248 women, 494 men) were retrospectively analyzed in this study. There were 559 (75.3%) patients in the high nutrition risk group, while 183 patients were in the low nutrition risk group. Overall hospital, 14-day and 28-day mortality rates were 32%, 12% and 22%, respectively. Patients’ most common comorbidities were sepsis, diabetes mellitus, congestive heart failure, chronic obstructive pulmonary disease, immunocompromised disorders, acute respiratory distress syndrome and liver cirrhosis. High nutrition risk patients were older, had higher APACHE II score, longer duration of ventilator dependency and longer duration of ICU and hospital stays. Although the hospital, 14-day and 28-day mortality rates were higher in the high nutrition risk group when compared to the low nutrition risk group, the difference did not reach statistically significance. The mean 7-days energy intake showed no difference between the high and low nutrition risk groups.

A logistic regression model was used to evaluate the associations of hospital (Table 2), 14-day (Table 3) and 28-day (Table 4) mortalities with energy intakes, both with and without adjustment for confounding factors. High mean energy intake was associated with lower hospital, 14-day and 28-day mortalities with and without adjustment for age, gender, BMI and APACHE II score in patients with high nutrition risk. However, the associations of hospital and 28-day mortality rates with mean energy intake were not observed in patients with low nutrition risk. We further examined the minimal amount of energy intake required to reduce the mortality rate in our critically ill patients by calculating odds ratio for each different value of energy intake. If patients with high nutrition risk could consume more than 800 kcal/day, the hospital, 14-day and 28-day mortality rates were significantly lower after adjusting for potential confounders, this trend was not observed in patients with low nutrition risk.

## 4. Discussion

Insufficient energy delivery has been shown to be associated with increased nosocomial infection, as well as longer durations of ventilator dependency, hospital and ICU stay [15,16,17,18]. Our previous study also revealed a similar finding that showed high energy intake could reduce the mortality rate in medical critically ill patients [19]. However, a limitation of our previous study was that patients’ nutritional status was not assessed [19]. Therefore, we did not know whether patients’ nutritional status would confound the association between energy intake and clinical outcomes. Not evaluating patients’ nutrition risk or not measuring their energy expenditure by indirect calorimetry, overfeeding during a critical illness might result in autophagy deficiency and organ damage [20]. The prognostic performance of NUTRIC or mNUTRIC score has been demonstrated to be significantly associated with mortality rates in Canadian, Asian, Dutch and Portuguese populations [8,11,12,21]. We thus examined the association between energy intake and mortality after identifying patients’ nutrition risk status using mNUTRIC score in the present study. Among our critically ill patients, 75.3% were at high nutrition risk. In comparison with other studies, this percentage was much higher than rates found in previous studies such as Mukhopadhyay et al. (45.4%) [11], de Vries et al. (61.4%) [12], Arabi et al. (42.3%) [14] and Mendes et al. (48.6%) [21]. Regardless of the distribution of high and low nutrition risk among studies, our findings were similar to those of other studies [9,11,13], in that high energy intake was associated with low hospital, 14-day and 28-day mortality rates in the critically ill patients with high nutrition risk. However, there was no difference in mortality between the permissive underfeeding and standard feeding groups in patients with either low or high nutrition risk in a post hoc analysis of the PermiT Study [14]. It is worth noting that both medical and surgical/trauma critically ill patients were combined in the study by Arabi et al. [14] and that surgical patients might receive less nutrition than medical patients [22]. To eliminate the heterogeneity of medical and surgical clinical condition, we only studied medical ICU patients in the present study. Since we had a high percentage of critically ill patients who were at high nutrition risk, early identification of critically ill patients’ nutrition risk is necessary and important for patients at high risk of adverse nutrition-related outcomes as they would benefit from receiving adequate nutrition [6].

In contrast to high nutrition risk patients, previous findings reported that energy intake and 28-day, 60-day, or 180-day mortality rates were not well correlated in patients with low nutrition risk [9,11,13,14]. Interestingly, in the present study an association between energy intake and 14-day mortality was found in our patients with low nutrition risk but there was no significant association with hospital mortality and 28-day mortality. In fact, 14-day mortality is considerably shorter than 28-day mortality and seems to be rarely assessed as a target clinical outcome in the clinical setting. The association between energy intake and 14-day mortality was not the primary focus of the present study and the amount of energy intake did not appear to be the main factor affecting clinical outcomes in patients with low nutrition risk. Further study is warranted to determine which factor might affect mortality in patients with low nutrition risk.

Although there is broad agreement that provision of adequate energy intake for critically ill patients is required to improve their clinical outcomes, the optimal amount of energy intake remains unclear. Trophic feeding, permissive under feeding and full caloric feeding are controversial with respect to risks or benefits of clinical outcomes in the ICU [23,24,25,26]. ASPEN and ESPEN guidelines suggested that energy requirement of 25–30 kcal/kg body weight/d [6] or 20–25 kcal/kg body weight [27] might be preferred for critically ill patients when using indirect calorimetry to measure energy expenditure is not available in the ICU. We further assessed the amount of energy intake required for high nutrition risk patients to reduce their mortality and found that patients with high nutrition risk who received at least 800 kcal/d had significantly reduced hospital, 14-day and 28-day mortality rates. However, Zusman et al. recommended that an intake of 70% of measured energy expenditure (~1361 kcal/day) could reduce the duration of ICU stay and ventilator use [28]. Although our reported amount of energy requirement did not precisely reflect our previously recommended amount (65% of energy requirement, ~934 kcal/day) [19] or that of Zusman et al. [28], the inconsistent amount was probably due to the fact that the patients’ nutritional status was not taken into consideration in the previous two studies [19,28]. Even though nutrition risk was considered in a previous trial [14], no mortality difference was observed between the permissive underfeeding and standard feeding groups in patients with either low (862 ± 308 vs. 1360 ± 491 kcal/day) or high (799 ± 277 vs. 1216 ± 420 kcal/day) nutrition risk. Regardless of patients’ nutritional status, previous studies indicated that higher caloric intake had no greater benefit in reducing gastrointestinal intolerance, infections, lengths of hospital and ICU stay and mortality rates when compared to lower caloric intake (300 ± 149 vs. 1418 ± 686 kcal/day in the study of Rice et al. [3]; 400 vs. 1300 kcal/day in a study by the National Heart, Lung and Blood Institute Acute Respiratory Distress Syndrome (ARDS) Clinical Trials Network [4]; and 835 ± 297 vs. 1299 ± 467 kcal/day in a study by Arabi et al. [5]). In summary, the results of the present and previous studies show that receiving at least 800 kcal/day seems to be the minimal energy amount for reducing mortality in high nutritional risk patients in the medical ICU.

The first strength of this study was that hospital mortality, 14-day and 28-day mortality was simultaneously assessed. Although this was a retrospective study, we collected a large sample size (742 medical critically ill patients). In addition, we were able to precisely recommend the minimal amount of energy intake patients with high nutrition risk require to improve their clinical outcomes. However, there were some limitations in this study. The first limitation was that this study employed a retrospective design and was conducted in a single center. Therefore, some confounding factors, such as albumin or prealbumin level, could not be completely collected and were not adjusted for the regression model. Other than total calories received by critically ill patients, adequate protein intake has also been emphasized in the critical care setting [6]. However, it was not possible to analyze the associations between macronutrient intakes (carbohydrate, lipid and protein) and clinical outcomes, as these nutrient intakes were not available in the patients’ medical chart records. Finally, the present results may not be generalizable to surgical critically ill patients since this study was conducted only in the medical ICU. A better nutritional approach for critically ill patients is to understand their acute changes of metabolism and complex combination of functional changes in multiple organ system related to nutritional status [29].

## 5. Conclusions

In conclusion, higher energy intake was associated with lower mortality in patients with high nutrition risk but this trend was not seen in patients with low nutrition risk. Although patients with low nutrition risk did not benefit from high energy intake, patients with high nutrition risk are suggested to consume at least 800 kcal/day in order to reduce their mortality rate in the medical ICU.

## Figures and Tables

**Table 1:** Demographic characteristics, clinical outcomes and energy intakes of all patients and patients with low or high nutritional risk in the medical intensive care unit.

Variables	All (*n* = 742)	Low Nutrition Risk (*n* = 183)	High Nutrition Risk (*n* = 559)
Age (year)	67.81 ± 16.22	55.59 ± 14.25 ^*^	71.80 ± 14.77
Gender (women/men)	248/494	60/123	188/371
Body mass index (kg/m^2^)	23.62 ± 4.83	23.72 ± 5.38	23.60 ± 4.64
Clinical outcomes			
Length of ventilatory dependency (day)	15.83 ± 14.88	13.17 ± 13.74 ^*^	16.70 ± 15.14
Length of ICU stay (day)	13.30 ± 8.15	11.37 ± 7.31 ^*^	13.93 ± 8.31
Length of hospital stay (day)	27.04 ± 19.35	25.80 ± 21.75 ^*^	27.44 ± 18.50
APACHE II score	26.99 ± 6.79	19.45 ± 4.98 ^*^	29.45 ± 5.32
mNUTRIC score	5.58 ± 1.80	3.07 ± 1.02 ^*^	6.40 ± 1.10
Mortality (*n*, %)			
Hospital mortality	237, 31.94%	35, 19.13%	202, 36.14%
14-day mortality	88, 11.86%	17, 9.28%	71, 12.70%
28-day mortality	163, 21.97%	26, 14.21%	137, 24.51%
Energy intakes			
Mean 7 day of energy intake (kcal/day)	692.68 ± 313.58	726.28 ± 342.39	681.69 ± 303.08
Comorbidities (*n*, %)			
Diabetes mellitus	289, 38.95%	40, 21.86%	249, 44.54%
Congestive heart failure	165, 22.24%	10, 5.46%	155, 27.73%
Liver cirrhosis	69, 9.30%	14, 7.65%	55, 9.84%
COPD	161, 21.70%	26, 14.21%	135, 24.15%
Immunocompromised disorders	126, 16.98%	22, 12.02%	104, 18.60%
Acute respiratory distress syndrome	89, 11.99%	15, 8.20%	74, 13.24%
Sepsis	373, 50.27%	57, 31.15%	316, 56.53%

Values are mean ± standard deviation. mNUTRIC, modified nutritional risk for critically ill patients; APACHE II, Acute Physiology and Chronic Health Evaluation II; COPD, chronic obstructive pulmonary disease. ^*^ Values are significantly different between low and high nutrition risk; *p* < 0.05.

**Table 2:** Adjusted odds ratios of hospital mortality in medical intensive care unit^1.^

	No Factors Adjusted for			Additional Factors Adjusted for Age, Gender, BMI			Additional Factors Adjusted for Age, Gender, BMI and APACHE II		
	OR	95% CI	*p*	OR	95% CI	*p*	OR	95% CI	*p*
Mean energy intake (kcal/day)									
All nutrition risk	0.999	0.998–0.999	<0.001	0.999	0.998–0.999	<0.001	0.999	0.999–1.000	0.003
High nutrition risk	0.999	0.998–0.999	<0.001	0.999	0.998–0.999	<0.001	0.999	0.998–1.000	0.002
Low nutrition risk	1.000	0.999–1.001	0.986	1.000	0.999–1.001	0.716	1.000	0.999–1.001	0.667
Mean energy intake (kcal/day)									
All nutrition risk									
>800 kcal/day	1			1			1		
≤800 kcal/day	1.982	1.414–2.780	<0.001	2.005	1.429–2.815	<0.001	1.569	1.100–2.240	0.013
High nutrition risk									
>800 kcal/day	1			1			1		
≤800 kcal/day	2.230	1.511–3.289	<0.001	2.134	1.441–3.159	<0.001	1.711	1.136–2.577	0.010
Low nutrition risk									
>800 kcal/day	1			1			1		
≤800 kcal/day	0.982	0.469–2.058	0.962	1.091	0.506–2.354	0.824	1.074	0.496–2.327	0.857

OR, odds ratio; BMI, body mass index. APACHE II, Acute Physiology and Chronic Health Evaluation II.

**Table 3:** Adjusted odds ratios of 14-day mortality in medical intensive care unit^1.^

	No Factors Adjusted for			Additional Factors Adjusted for Age, Gender, BMI			Additional Factors Adjusted for Age, Gender, BMI and APACHE II		
	OR	95% CI	*p*	OR	95% CI	*p*	OR	95% CI	*p*
Mean energy intake (kcal/day)									
All nutrition risk	0.997	0.996–0.997	<0.001	0.997	0.996–0.997	<0.001	0.997	0.996–0.998	<0.001
High nutrition risk	0.996	0.995–0.997	<0.001	0.996	0.995–0.997	<0.001	0.996	0.995–0.997	<0.001
Low nutrition risk	0.998	0.997–1.000	0.028	0.998	0.996–1.000	0.013	0.998	0.996–0.999	0.011
Mean energy intake (kcal/day)									
All nutrition risk									
>800 kcal/day	1			1			1		
≤800 kcal/day	4.233	2.258–7.937	<0.001	4.210		<0.001	3.459	1.826–6.553	<0.001
High nutrition risk					2.244–7.901				
>800 kcal/day	1			1			1		
≤800 kcal/day	5.534	2.483–12.333	<0.001	5.346	2.390–11.958	<0.001	4.341	1.918–9.823	<0.001
Low nutrition risk									
>800 kcal/day	1			1			1		
≤800 kcal/day	2.127	0.717–6.307	0.174	2.398	0.784–7.336	0.125	2.368	0.773–7.257	0.131

OR, odds ratio; BMI, body mass index. APACHE II, Acute Physiology and Chronic Health Evaluation II.

**Table 4:** Adjusted odds ratios of 28-day mortality in medical intensive care unit^1.^

	No Factors Adjusted for			Additional Factors Adjusted for Age, Gender, BMI			Additional Factors Adjusted for Age, Gender, BMI and APACHE II		
	OR	95% CI	*p*	OR	95% CI	*p*	OR	95% CI	*p*
Mean energy intake (kcal/day)									
All nutrition risk	0.998	0.998–0.999	<0.001	0.998	0.998–0.999	<0.001	0.998	0.998–0.999	<0.001
High nutrition risk	0.998	0.997–0.999	<0.001	0.998	0.997–0.999	<0.001	0.998	0.998–0.999	<0.001
Low nutrition risk	0.999	0.998 –1.000	0.171	0.999	0.997–1.000	0.077	0.999	0.997–1.000	0.064
Mean energy intake (kcal/day)									
All nutrition risk									
>800 kcal/day	1			1			1		
≤800 kcal/day	2.263	1.518–3.373	<0.001	2.268	1.520–3.383	<0.001	1.803	1.192–2.728	0.005
High nutrition risk									
>800 kcal/day	1			1			1		
≤800 kcal/day	2.450	1.548–3.878	<0.001	2.297	1.445–3.652	<0.001	1.850	1.146–2.988	0.012
Low nutrition risk									
>800 kcal/day	1			1			1		
≤800 kcal/day	1.390	0.594–3.253	0.447	1.572	0.647–3.821	0.318	1.549	0.635–3.775	0.336

OR, odds ratio; BMI, body mass index. APACHE II, Acute Physiology and Chronic Health Evaluation II.

## Abbreviations

The following abbreviations are used in this manuscript:

APACHE II	acute physiology and chronic health evaluation II
BMI	body mass index
ICU	intensive care unit
IL-6	interleukin-6
NUTRIC	Nutrition Risk in the Critically Ill
TCVGH	Taichung Veterans General Hospital
